# The Enigma of Tripeptidyl-Peptidase II: Dual Roles in Housekeeping and Stress

**DOI:** 10.1155/2010/128478

**Published:** 2010-08-18

**Authors:** Giulio Preta, Rainier de Klark, Riccardo Gavioli, Rickard Glas

**Affiliations:** ^1^Center for Molecular Medicine (CMM), Department of Medicine, Karolinska Institute, Karolinska University Hospital, 171 76 Stockholm, Sweden; ^2^Department of Biochemistry and Molecular Biology, University of Ferrara, 44100 Ferrara, Italy

## Abstract

The tripeptidyl-peptidase II complex consists of repeated 138 kDa subunits, assembled into two twisted strands that form a high molecular weight complex (>5 MDa). TPPII, like many other cytosolic peptidases, plays a role in the ubiquitin-proteasome pathway downstream of the proteasome as well as in the production and destruction of MHC class I antigens and degradation of neuropeptides. Tripeptidyl-peptidase II activity is increased in cells with an increased demand for protein degradation, but whether degradation of cytosolic peptides is the only cell biological role for TPPII has remained unclear. Recent data indicated that TPPII translocates into the nucleus to control DNA damage responses in malignant cells, supporting that cytosolic “housekeeping peptidases” may have additional roles in cell biology, besides their contribution to protein turnover. Overall, TPPII has an emerging importance in several cancer-related fields, such as metabolism, cell death control, and control of genome integrity; roles that are not understood in detail. The present paper reviews the cell biology of TPPII and discusses distinct roles for TPPII in the nucleus and cytosol.

## 1. Introduction

Tripeptidyl-peptidase II (TPPII), discovered in 1983, is an aminopeptidase that removes tripeptides from the free N-terminus of oligopeptides and is regarded as a housekeeping enzyme of eukaryotic cells [1–3]. TPPII is present in all examined higher species, including invertebrates (e.g., *C. Elegans* and *D. Melanogaster)*, is absent in certain protozoa and many fungal species, but a TPPII orthologue is also present in *S. Pombe * [4, 5]. It is the largest cytosolic peptidase complex of mammalian cells (>5 MDa), and is composed of two twisted strands of stacked TPPII dimers, composed of unique 138-kDa subunits [6]. The N-terminal domain of TPPII contains a serine peptidase site, with a classical catalytic triad of serine peptidases (Ser-449-His-264-Asp-44) [7]. The only demonstrated method of activation of this enzyme is through complex formation, which could be the principal means by which the enzymatic activity of TPPII is regulated [8–10]. TPPII is known to participate in protein turnover, presumably in concert with the proteasome and other exopeptidases [11–14], and possesses the ability to degrade oligopeptides >15 amino acids long [15]. It cleaves efficiently after hydrophobic residues, fails to cleave before or after proline residues, and displays endopeptidase activity by cleaving after lysine residues, although this activity is weak when compared to its exopeptidase function [3, 12].

Protein degradation is critical for many cellular functions and alterations in the metabolism of proteins involved in the cell cycle and apoptosis may result in uncontrolled cell division, which can lead then to malignant transformation. The overexpression of TPPII observed in a specific tumor as Burkitt's lymphoma [14], where an impaired proteasome function is also present, points out the essential role that this peptidase may play in tumor metabolism. TPPII may increase the rate by which free amino acids are produced during protein breakdown, a role shared with many other cytosolic and nuclear peptidases. Also, like other cytosolic peptidases, TPPII influences MHC class I processing; usually through N-terminal trimming or destruction of MHC class I epitopes [15–21]. Degradation of peptide hormones is another common theme, for example, for TPPII, Thimet oligopeptidase (TOP), and Dipeptidyl-peptidase-4 (DPP-4) [2, 22, 23]. TPPII degrades CCK-8, an octapeptide that is the main form of CCK in mammalian brain [2, 24].

So, is the cell biological role of TPPII restricted to protein turnover and degradation of cytosolic peptides, or does this very large peptidase complex also have other types of more specialized functions? A regulatory role has previously been reported for DPP-4, a ubiquitously expressed transmembrane glycoprotein with known substrates that include several growth factors, neuropeptides, and chemokines. DPP-4 belongs to the S28 Serine protease family, and inhibitors of this peptidase are now in clinical practice for treatment of type 2 diabetes [23]. The expression and localization of TPPII is altered in response to several forms of stress, which suggested a possible role for TPPII in signaling. In several cases, remarkable phenotypes are observed upon altered expression of TPPII, in animals or cell lines; phenotypes of unclear causes (Table 1) [25, 34, 36, 37, 42, 48]. Herein, we review some of the main reported aspects of TPPII biology in housekeeping versus signaling in response to cellular stress.

## 2. TPPII as a Generator of MHC Class I-Binding Peptides

**Figure 1:**
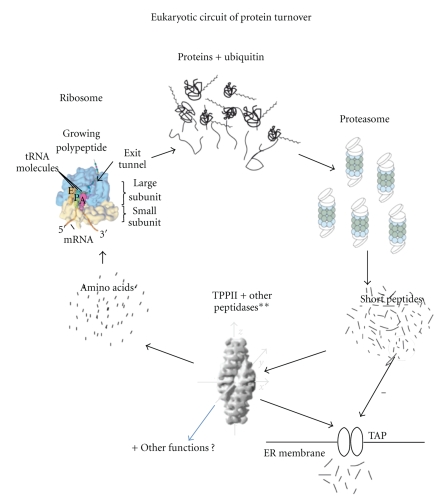
The eucaryotic circuit of protein turnover—recycling of proteins into amino acid building blocks. Amino acids are incorporated into proteins that are subsequently degraded by the ubiquitin-proteasome pathway into peptides, of which a small minority are sampled by the MHC class I processing pathway, in vertebrates. Downstream of proteasomal protein degradation, most peptides are degraded into shorter peptides and amino acids by cytosolic peptidases, a step function that is highly redundant due to the presence of many different cytosolic peptidases. Note that a few cytosolic peptides are not proteasomal degradation products, but peptide hormones. **Examples of cytosolic peptidases: Tripeptidyl-peptidase II (TPPII), Bleomycin Hydrolase (BLMH), Thimet oligopeptidase (TOP), and Puromycin-sensitive aminopeptidase (PSA); see also Table 1 where phenotypes induced by their modulation in cells and knockout animals are displayed. The proteasome is present in all eucaryotes (as well as in archaebacteria); several oligopeptidases also show a high level of conservation. TPPII orthologues is expressed in *D. Melanogaster, C. Elegans, A. Thaliana*; as well as in *S. Pombe*.

Most investigations of TPPII in protein turnover have focused on its role in the generation of MHC class I-bound peptides, a common way to probe the specificity of cytosolic proteolysis. Peptides bound to MHC class I molecules are derived from the pool of peptides that originate from degraded cytosolic proteins, which are made available for assembly with MHC class I through TAP1/2-mediated translocation across the ER-membrane (Figure 1). Although proteasomes are essential in the generation of the C terminus of most peptides that bind MHC class I molecules, proteasomal inhibitors have a negligible effect on a subset of MHC class I epitopes. In particular, the generation of epitopes with a lysine residue as the C-terminal anchor is scarcely altered by proteasome inhibition (epitopes of HLA-A3, -A11, and -B35) [52]. During the ongoing search for additional peptidase activities in the generation of MHC class I-bound peptides several epitopes generated by TPPII have been indicated, for example, HIV-1 Nef 73-82; an immunodominant HLA-A3/A11 epitope (Table 2) [16]. However, this is not exclusive to lysine anchor motifs, as recently observed in two HLA-A2-restricted EBV-derived epitopes of LMP1 (Table 2) [19], and the H-2K^d^-restricted Influenza virus epitope NP147-155, although another report failed to confirm the latter finding [20, 49]. A dependence on TPPII in the presence of proteasomal inhibition was found for the *L. monocytogenes-*derived CD8^+^ T cell H-2K^d^ epitope LLO91-99 whereas another epitope (p60 449-457) required the activity of both TPPII and the proteasome in infected macrophages [21]. Moreover, two further Listeria epitopes were found to be unaffected by TPPII inhibition (p60-217-225, p60-476-484) [21]. Studies using extended versions of peptide epitopes have revealed that TPPII can contribute to trimming of their N-terminals, as shown for the H-2K^b^-restricted ovalbumin epitope SIINFEKL and RU-1, a renal carcinoma tumour antigen [50, 51]. Nevertheless, presentation of SIINFEKL from intact OVA protein was only modestly reduced by inhibition of TPPII expression [51]. Furthermore, pharmacological TPPII inhibition failed to inhibit the processing of six different lymphocytic choriomeningitis virus- (LCMV-) derived T cell epitopes, although a small enhancement of antigen processing was noted in some cases [26]. Similar results were found for SIINFEKL and an H-2L^d^-restricted murine cytomegalovirus (MCMV) epitope (pp89/168-176) [26]. Moreover, experiments using dendritic cells (DCs) from TPPII−/− knockout mice revealed normal presentation of several LCMV epitopes (GP33-41, GP276-286, NP396-404), and the ovalbumin epitope SIINFEKL was generated even more efficiently by TPPII−/− dendritic cells (DCs) than by their control counterparts [18]. In addition, using TPPII gene-trapped mice, a lack of significant difference compared to control mice (TPPII wild type) was seen in the response to four epitopes from LCMV (Table 2; >90% reduction in TPPII expression) [27]. The presentation of peptide precursors with long N-terminal extensions in TPPII gene-trapped embryonic fibroblasts was modestly reduced *in vitro* whereas the presentation of full-length OVA protein was unaltered. Further, an equivalent CD8^+^ T cell response was induced *in vivo* in wild type and TPPII-deficient mice immunized with recombinant lentiviral or *vaccinia* vectors regardless of the extension at the N-terminus of SIINFEKL [27]. Thus, only a minority of examined MHC class I epitopes depend on TPPII for their generation.

Does TPPII influence the overall rate of production of MHC class I ligands? Such a role has been suggested by experiments using a specific TPPII inhibitor to block the cell surface expression of MHC class I almost as efficiently as a proteasomal inhibitor [15]. However, using both RNA interference and pharmacological inhibition by butabindide, Marcilla et al. obtained no effect on peptide loading for HLA-B27, -A3, -A68, and B14-expressing cells [53]. Furthermore, York et al. demonstrated that elimination of TPPII from human cells using RNAi does not decrease the overall supply of peptides to MHC class I molecules [51]. In addition the surface expression of MHC class I molecules (H-2K^b^, D^b^) on several cell types from TPPII−/− gene-deficient mice as well as TPPII gene-trapped mice was even slightly increased, indicating a modest destructive role in MHC class I antigen production [18, 27]. A potential role for TPPII-mediated destruction of a specific MHC class I epitope has also been observed in colon carcinoma cells, when presentation of an HLA-A2-restricted epitope of survivin was examined [28].

In conclusion, studies have demonstrated a contribution of TPPII in the processing of selected antigens, but its overall importance in the generation of MHC class I-bound peptide pools appears to be minor [54]. TPPII influences production of MHC class I ligands; (1) downstream of the 26S proteasome, in its main nonredundant role, by N-terminal trimming of longer (>15 a.a.) peptide intermediates generated by the proteasome. However, this role may have limited importance for the MHC class I pathway in live cells, since the majority of peptides that the proteasome produces are shorter than this [51]. TPPII can also act; (2) in the direct generation of a minority of MHC class I epitopes, for example, those with basic C-termini, although the number of known epitopes generated by TPPII independently of the proteasome is very small (Table 2). No cytosolic peptidase of major importance for trimming of MHC class I antigens has yet been elucidated, instead most peptides appear to be N-terminally trimmed by ER aminopeptidase 1 (ERAP1) [55–57]. The main influence of TPPII on MHC class I processing is likely to be cytosolic destruction of epitopes, since data from two different types of TPPII-deficient mice show increased MHC class I expression [18, 27]. A destructive influence on MHC class I antigen processing has also been reported for Puromycin-sensitive aminopeptidase (PSA) and Thimet Oligopeptidase (TOP) [39, 45]. Sequencing of the MHC class I-bound repertoire in TPPII−/− cells may reveal more about subsets of MHC class I antigens that are negatively or positively influenced by TPPII expression.

## 3. TPPII in Contribution to Protein Metabolism

**Figure 2:**
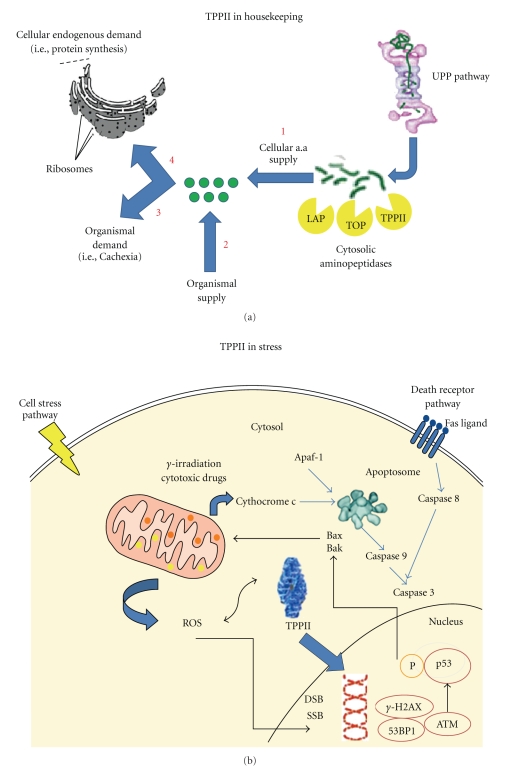
Distinct functions for TPPII in the nucleus and cytosol. (a) TPPII contributes to protein turnover in concert with components of the ubiquitin-proteasome pathway and other cytosolic peptidases. The expression and activity of TPPII is influenced by the following. (1) The cellular recycling of amino acids from protein building blocks, that is, through degradation of proteins by the ubiquitin-proteasome pathway, inhibited recycling leads to increased activity of TPPII [11, 12, 14]. (2) The external supply of amino acids. Dilution of cell culture medium in PBS increases TPPII levels [25]. (3) The organismal demand for amino acids. An increased demand, as observed in cachexia also increases TPPII activity [29, 30]. (4) Endogenous demand for amino acids; a factor that potentially increases TPPII; remains to be tested. (b) In response to ROS production and DNA damage, TPPII shifts its localization from cytosolic to nuclear. This may contribute to the DNA damage response and apoptosis triggering, a model that is under debate. The cytosolic-nuclear shift of TPPII may create a link between mitochondrial respiration and DNA damage signaling in the nucleus.

The main housekeeping role of cytosolic peptidases is to provide sufficient free amino acids for cellular needs in protein synthesis. Several studies have addressed the expression of TPPII in situations of increased demand for amino acids, that is, focusing on the absolute levels of TPPII expression and/or activity rather than its cleavage specificity in sampling of MHC class I antigens. Conditions of increased proteasomal protein degradation and demand for amino acids due to tumor-induced cachexia in rats was found to cause enhanced activity of TPPII in muscles [29]. Furthermore, a glucocorticoid-dependent muscle wasting correlates with high TPPII activity during sepsis [30]. In addition, increased expression of TPPII has been observed in EL-4 lymphoma cells grown in cell culture medium diluted with PBS [25]. In these studies, the increased expression of TPPII correlated with an increased demand for the amino acid production by the ubiquitin-proteasome pathway (UPP), due to either increased external demand or decreased supply of amino acids in the medium. Given that the UPP recycles protein building blocks into free amino acids, the question of how TPPII responds to a decrease in proteasomal activity may be raised [58]. Such experiments have been performed, by adapting *in vitro* cell lines to the presence of covalently acting proteasomal inhibitors (NLVS, ZL_3_VS, or Lactacystin). Such adapted cells displayed inhibited activity of the proteasome and upregulation of TPPII [11, 12, 49]. In addition, a similar phenotype (low proteasomal activity and high TPPII activity) was present in most Burkitt's lymphoma cells (BLs); where the oncogene c-Myc was strongly upregulated [14]. The c-Myc protein is stabilized in a number of BL cell lines, suggesting that defective UPS c-Myc proteolysis may play a role in the lymphomagenesis. It should however be noted that TPPII is not upregulated in several types malignant cells adapted to the presence of Bortezomib (a boronic acid proteasome inhibitor) [31]. Nonetheless, these findings are consistent with TPPII as a housekeeping enzyme in protein turnover with upregulation either during increased amino acid demand or in response to inhibited endogenous supply by the UPP (Figure 2(a)).

In addition to the role of TPPII in oligopeptide degradation downstream of the proteasome, the finding of increased TPPII activity in combination with blocked chymotryptic proteasomal activity in NLVS-adapted EL-4 cells (EL-4ad, growing in either NLVS or lactacystin) initiated a discussion as to whether TPPII could contribute to protein turnover in the absence of the proteasome [11, 12]. This theory was lent further weight by that stable overexpression of TPPII in an EL-4 transfectant allowed resistance to NLVS-treatment, as well as the finding of reduced proteasomal activity in BLs [13, 14]. However, it should be mentioned that EL-4ad, BLs and EL-4.pcDNA3-TPPII cells (over-expressing TPPII) still had some proteasomal activity, and that they were probably still dependent on this activity [14, 15, 59]. Present data support a housekeeping role for TPPII mainly downstream of the proteasomal protein degradation, and a TPPII-mediated degradation of protein substrates has thus far not been demonstrated. Nonetheless, a TPPII-mediated contribution to protein turnover in parallel with the proteasome cannot be excluded, for example, through the degradation of unfolded polypeptides.

## 4. TPPII in Apoptosis Regulation and Genetic Stability

In addition to a housekeeping function in protein turnover, with the consequent generation of MHC class I ligands, several reports have indicated other more specialized roles for TPPII. The enigma discussed herein is common to many other cytosolic peptidases, characterized by being unclear; their physiological roles and the peculiar phenotypes seen in cell lines and mice upon modulation of their expression (Table 1) [25, 34, 36, 37, 42, 48]. An altered expression of peptide hormones may have a role in the phenotypes of peptidase knockout animals, but is less likely to affect cell lines *in vitro*. Another mechanism for such observations may be an altered intracellular concentration of short peptides that modulate protein-protein interactions, that is, an indirect consequence of the contribution to protein turnover [46, 60]. Alternatively, they may have direct roles in signaling, by targeting a pool of natural substrates (as reported for DPP-4, in cleavage of substrates at the cell membrane [23]).

Inhibited apoptosis-sensitivity of EL-4ad cells correlated with increased expression of antiapoptotic proteins (IAPs), whose translation are turned on during stress through IRES (internal ribosomal entry site) sequences in their mRNAs [25, 58]. IAPs are endogenous inhibitors of caspases that are degraded by the proteasome in response to cytochrome C release (by their internal RING-domain, i.e., a mechanism of autoubiquitination) [61]. IAPs caused resistance to apoptosis in EL-4ad cells, and increased IAP expression was also present in transfected cell lines with stable overexpression of TPPII [25, 34]. This overexpression of IAP was connected to a compromised proteasomal activity, in presence of an overexpression of TPPII, which anyway was unable itself to degrade this class of proteins. Stabilization of IAPs may point to the hyphotesis that TPPII contributes to tumorigenicity by sustaining protein turnover without influencing degradation of antiapoptotic and proliferative proteins [25]. The expression of IAPs was variable between TPPII-transfected cell lines, with high expression of XIAP and cIAP1 in EL-4.pcDNA3-TPPII whereas HEK-293-TPPII cells expressed mainly high levels of c-IAP2. Furthermore, overexpression of TPPII by transfection into several cell lines causes evasion of mitotic arrest, as well as the formation of polyploid cells, possibly through abnormal centrosome regulation [34, 35]. Interestingly, the ectopic expression of cIAP1 perturbs mitotic control and promotes the formation of polyploid cells [62]. Previous findings in the patent literature support this evidence, showing that overexpression of TPPII in tumor cells creates genetic instability by causing centrosomal duplication errors [63]. In addition, in a study where WT and TPPII deficient fibroblasts were used NF-*κ*B activation upon TNF addition was defective in TPPII-deficient fibroblasts, as seen by the reduced nuclear accumulation of NF-*κ*B p65 [37]. Constitutive activation of the NF-*κ*B pathway is involved in different forms of cancer such as leukemia, lymphoma, colon cancer, and ovarian cancer. Further, previous data indicated that also PSA is associated with the mitotic apparatus and is involved in mitotic control [64]. Thus, TPPII expression has a substantial impact on IAP expression, programmed cell death and mitotic errors, but how TPPII controls these phenotypes still remains to be elucidated.

## 5. Nuclear Translocation of TPPII in Response to ROS and DNA Damage

Recent reports have studied the localization of TPPII in response to *γ*-irradiation, one demonstrated a translocation of TPPII into the nucleus [33], while the other authors did not observe this finding [65]. In the former study [33], TPPII accumulated in the nucleus in 7 out of 9 *γ*-irradiated tumor cell lines, and also in response to treatment with etoposide. This event was dependent on the presence of reactive oxygen species (ROSs) produced by mitochondria, since nuclear expression of TPPII was blocked by N-acetyl-cystein (an anti-oxidant) as well as rotenone (an inhibitor of mitochondrial respiration) [33]. Most cellular ROS are produced by the mitochondria, and ROS represent a constant hazard for the cell, most notably to the genome [66]. Such ROS have a strong influence on the DNA damage response; for example, in p53 expression in response to *γ*-irradiation of leukaemic cells [67, 68]. The other authors, [65] used both immunofluorescence and cytosolic/nuclear fractionation to investigate the localization of TPPII but nuclear translocation was not detected in EL4, COS cells, and transformed fibroblasts after different doses of *γ*-irradiation. An affinity-purified rabbit serum against a synthetic peptide corresponding to the NH_2_ terminus of TPPII was used for western and immunocitochemistry analysis. ROS levels, and the requirement for high cell densities, in experiments studying nuclear TPPII localization, may be the most likely reason for the observed experimental discrepancies [33, 65].

A role for TPPII in the nuclear expression of p53 in response to DNA damage was suggested, but this is currently controversial. Nuclear TPPII expression correlated with p53 expression in lymphoma cells [33]; for example, a short peptide-derived inhibitor that inhibited nuclear accumulation of TPPII also inhibited nuclear expression of p53 [33]. However, c-myc transformed fibroblasts, as well as normal cells, from gene-deficient TPPII−/− mice have normal p53 protein levels and functional p53 signaling [65, 69]. These differences may depend upon the role played by TPPII in different malignant cell types, but the potential role for p53 in the DNA damage is at this point uncertain. A putative role for TPPII in nuclear regulation of DNA damage responses is illustrated in Figure 2(b), alongside the housekeeping role for TPPII in Figure 2(a). Proteins with roles in DNA damage responses, that control cell cycle arrest and DNA repair, have attracted great interest as putative targets in cancer treatment as sensitizers to classical cancer therapy, such as *γ*-irradiation. In this respect, modulation of TPPII function may be interesting in cancer therapy [33, 65].

Several previously studied housekeeping proteins were found to acquire additional roles in response to stress. Mitochondrial respiration is linked to DNA damage responses through the dislocation of cytochrome C from their inter-membrane space into the cytosol, the classical trigger of caspase 9-activation and apoptosis. Furthermore, nuclear release of linker histone H1.2, into the cytosol has been reported as essential for mitochondrial cytochrome C release [70]. Thus, in addition to its housekeeping role in control of chromatin topology, H1.2 is also a signaling protein in *γ*-irradiated cells. Another housekeeping protein translocates in the reverse direction; apoptosis-inducing factor (AIF), a flavoprotein important for mitochondrial function, is shifted from the mitochondrial intermembrane space to the nucleus, where it promotes caspase-independent apoptosis [44]. The dual roles of these housekeeping proteins may reflect the necessity to disrupt basal metabolism in response to hostile conditions.

## 6. Observations in TPPII-Deficient Animals

TPPII is present in the genome of most studied eukaryotes, although it is absent from certain species of protozoa and fungi [4]. Suppressed expression of TPPII orthologues in *C. Elegans* (siRNA) [36], *A. Thaliana* (T-DNA mutant) [71], and *S. Pombe* (gene deletion) [4] had no obvious effects on viability. Three investigations into TPPII-deficient mice have recently been carried out; knock-out mice homozygotic for TPPII−/− [37], gene-trapped mouse that failed to produce TPPII homozygous mutants due to early embryonic lethality (before day 9,5) [36], and one study of a homozygously mutant TPPII gene-trapped animal with >90% reduced expression of TPPII [27]. In TPPII−/− mice declining levels of thymocytes and peripheral CD8^+^ cells was present in mice >12 months of age. Further, TPPII−/− displayed splenomegaly, increased granulocyte numbers in the spleen and extramedullary hematopoiesis [37]. In addition, deregulation of NF-*κ*B activation and premature senescence were seen in TPPII−/− fibroblasts cultured *in vitro*. Senescence may be regarded as a response to chronic DNA damage, which is interesting when considering TPPII in relation to DNA damage responses [72]. An alternative explanation for such senescence could be failure to activate NF-*κ*B in association with TPPII deficiency, as observed in TPPII−/− fibroblasts, since altered NF-*κ*B activation has repeatedly been observed alongside cellular senescence and systemic aging [73]. In the study by McKay et al., which failed to result in gene-trapped TPPII-mutant homozygotes, a phenotype was observed for TPPII-mutant heterozygotes, as demonstrated by decreased fat cell formation [36]. This study indicated an evolutionarily conserved role for TPPII, since *C. elegans* with downregulated TPPII expression also showed reduced fat storage, but this effect is unrelated to expression of the N-terminal region of TPPII, containing its peptidase site. The study of gene-trapped homozygously TPPII-mutant mice, produced by Kawahara et al., showed a reduction of TPPII mRNA expression of >90%, resulting in apparently healthy animals [27]. In these TPPII-deficient mice, there was an absence of observations made in TPPII−/− knockout mice, both with respect to hematopoietic symptoms, and abnormal histology [27]. From reviewing the literature it is clear that mutating the TPPII gene, causing a reduced expression, results in diverse effects. It is not clear why there is such major difference in the various TPPII mutant mice produced, and whether the difference in observed phenotypes among TPPII-deficient mice depend on animal housing or other parameters. It should be pointed out that TPPII-mutant mice produced by gene-trap may have incomplete inhibition of TPPII expression, or other effects of the genetic insert; compared to mice produced by homologous recombination. In conclusion, all reports of MHC class I processing in TPPII-mutant mice indicated a minor role for this peptidase in the production of MHC class I antigens (discussed in the previous section). Further, there are observations on aberrant cells death control within the hematopoietic system (that differ between gene-trapped and knock-out animals), and a further discussion must await more data from these animals [27, 36, 37].

## 7. Conclusion

In this paper, we have reviewed the cell biology of TPPII, and discussed distinct housekeeping versus stress-induced functions of TPPII. This discussion may in part be applicable to other evolutionary conserved peptidases that also have largely unknown cell biological roles. One example is Bleomycin hydrolase (the mammalian homologue of yeast Gal6), a peptidase complex (6 × 40 kDa), which is upregulated in some cancer cells and with an ability to bind double- and single-stranded DNA [42]. The link between TPPII and responses to stress, in particular to DNA damage, may be of considerable interest in cancer biology.

TPPII is upregulated during conditions of both external and internal depletion of amino acids, as observed from starvation or inhibited recycling by UPP inhibition, as well as increased organismal demand for amino acids observed in cachexia. The role for TPPII in processing of MHC class I antigens appears limited (at least compared to that of the proteasome), and more devoted to destruction than generation of epitopes, as also observed for TOP and PSA [39, 45]. Further, TPPII degrades CCK; in line with the observed degradation of peptide hormones by DPP-4 and TOP [2, 22, 23]. In intracellular signaling, the perturbation of apoptosis control and genetic stability mediated by the overexpression of TPPII may be due to increased IAP expression, but other regulatory alterations caused by TPPII are not excluded. The cytosolic-nuclear translocation of TPPII into the nucleus in several types of malignant cells exposed to genotoxic stress has indicated a link to DNA damage responses (Figure 2(b)). Specific substrates or interaction partners of TPPII may yield a deeper insight into how this evolutionary conserved peptidase complex contributes to intracellular signaling.

## Figures and Tables

**Table 1:** Phenotypes/pathways influenced by altered expression of selected cytosolic peptidase*s* ^&^.

Pathway/phenomena^#^	Model	Ref.
*Tripeptidyl-peptidase II (TPPII)*		
Neuropeptide degradation	cells	[2]
MHC class I processing*	cells	[15–21, 25–28]
	KO/GTmice	[18, 27]
Muscle wasting/cachexia	rats	[29, 30]
Resistance to proteasome inh.*	cells	[11–14, 31]
Apoptosis progression*	cells	[25]
DNA damage response*	cells	[32, 33]
G2/M progression	cells	[34, 35]
Fat formation	KO (+/−) mice	[36]
Hematopoietic homeostasis*	KO/GT mice	[27, 37]
Organismal lifespan	KO mice	[37]

*Thimet Oligopeptidase (TOP)*		
Neuropeptide degradation	cells	[22]
APP processing^⋆^	cells	[38]
MHC class I processing	cells	[39]

*Bleomycin Hydrolase*		
APP processing^⋆^	cells	[40]
MHC class I processing*	cells	[41]
Bleomycin resistance	KO mice	[42]
Dermal maturation	KO mice	[42]
Astrogliosis	KO mice	[43]
Cognitive functions	KO mice	[43]
Homocysteine metabolism	cells	[44]

*Puromycin-sensitive aminopeptidase *		
*(PSA)*		
MHC class I processing*	cells/KO mice	[45]
G2/M progression	cells	[46]
Growth, reproduction	KO/GT mice	[47]
Anxiety, pain control	GT mice	[47]

^&^Phenotypes and pathways studied in live mammalian cells or in mice.

^#^Both known pathways and phenomena, several of unknown cause, are included in this list. Thereby, it is possible that some phenomena are the result of some of the known pathways, although the link has not been made. For example, it is unclear whether degradation of neuropeptides contributes to any of the signs in TPPII−/− mice.

*The contribution of the peptidase is debated, since reports show both a presence and absence of a role in the phenomenon.

^@^KO: knock-out mice; GT gene-trapped mice.

^⋆^ *A*PP: amyloid precursor protein.

**Table 2:** Reported TPPII-dependency in processing of MHC class I-bound epitopes in live cells.

Source	MHC class I	Sequence	Cell type	In vivo^Ψ^	Ref.^#Θ^
Requirement for TPPII*					
HIV-1 Nef_73-82_	HLA-A3/A11	QVPLRPMTYK^&@^	DC, LCLs	—	[16]
EBV-LMP1	HLA-A2	YLLEMLRWL^&@^	COS cells, LCLs	—	[19]
EBV-LMP1	HLA-A2	YLQQNWWTL^&@^	COS cells, LCLs	—	[19]
Influenza NP_147-155_	H-2K^d^	(TYQRTRALV)^&@#^	HEK-293, L cells	—	[20, 49]^#^
Listeria-LLO_91-99_	H-2K^d^	GYKDGNEYI^&Δ^	J774, BMM	—	[21]
Listeria-p60_449-457_	H-2K^d^	IYVGNGQMI^&^	J774, BMM	—	[21]
RU1_34-42_	HLA-B51	VPYGSFKHV^&^	BB-64 Renal carcinoma	—	[50] ^Θ^

*No requirement for TPPII*					
Listeria-p60_217-225_	H-2K^d^	KYGVSVQDI^&^	BMM	—	[21]
Listeria-p60_476-484_	H-2K^d^	KYLVGFGRV^&^	BMM	—	[21]
OVA	H-2K^b^	SIINFEKL^&§^	BMC-2, TPPII−/− DC	TPPII−/− GT^⋆^	[18, 26, 27, 51]
LCMV NP_118-126_	H-2L^d^	RPQASGVYM^&^	J774	—	[26]
LCMV GP_33-41_	H-2K^b^/D^b^	KAVYNFATC^&§^	BMC-2, HEK-293, TPPII−/− DC	TPPII−/−, GT	[18, 26, 27]
LCMV GP_276-286_	H-2D^b^	SGVENPGGYCL^&§^	BMC-2, HEK-293, TPPII−/− DC	TPPII−/−, GT	[18, 26, 27]
LCMV NP_396-404_	H-2D^b^	FQPQNGQFI^&§^	BMC-2, HEK-293, TPPII−/− DC	TPPII−/−, GT	[18, 26, 27]
LCMV NP_205-212_	H-2K^b^	YTVKYPNL^&§^	BMC-2	GT	[26, 27]
LCMV GP_92-101_	H-2D^b^	CSANNSHHYI^&^	BMC-2, HEK-293	—	[26]
MCMV- pp89_168-176_	H-2L^d^	YPHFMPTNL^&^	L cells	—	[26]

*Epitope destruction by TPPII*					
Survivin	HLA-A2	ELTLGEFLKL^&^	Colon carcinoma	—	[28] ^Θ^

*The effect considered has to be at least partial in the processing of an epitope in live cells.

^Ψ^ *I* *n vivo* dependence on TPPII for generation of a CTL response against epitope, the column contains the animal used.

^#^The requirement for TPPII in the processing of this epitope is debated.

^Θ^Evidence in some reports is based only on AAF-CMK, an inhibitor that is however not TPPII-specific.

Evidence base: catalytic inhibitor—&; siRNA—@; gene-deficient cells—§.

^⋆^GT: Gene-trapped TPPII-deficient mouse.

^Δ^Only in the presence of proteasomal inhibitor.

Cells: Dendritic cells, COS: Chinese Hamster Ovary cells, LCL: Lymphoblastoid cells, HEK293: Human embryonic kidney cells, BMC-2 and J-774 - macrophage cell lines, BMM: Bone-marrow macrophages.
